# *Caenorhabditis* microbiota: worm guts get populated

**DOI:** 10.1186/s12915-016-0260-7

**Published:** 2016-05-09

**Authors:** Laura C. Clark, Jonathan Hodgkin

**Affiliations:** Department of Biochemistry, University of Oxford, South Parks Road, Oxford, OX1 3QU UK

## Abstract

Until recently, almost nothing has been known about the natural microbiota of the model nematode *Caenorhabditis elegans*. Reporting their research in *BMC Biology*, Dirksen and colleagues describe the first sequencing effort to characterize the gut microbiota of environmentally isolated *C. elegans* and the related taxa *Caenorhabditis briggsae* and *Caenorhabditis remanei* In contrast to the monoxenic, microbiota-free cultures that are studied in hundreds of laboratories, it appears that natural populations of *Caenorhabditis* harbor distinct microbiotas.

## A model without a microbiota

*Caenorhabditis elegans* has been used as a model organism since the 1940s and came to prominence in the 1970s with the work of Sydney Brenner, who used *C. elegans* to investigate neurobiology and development [1]. Brenner received the worms that were to found the N2 experimental strain as an axenic culture (one in which no other organisms are present) and subsequently propagated them on a single strain of *Escherichia coli* (a monoxenic culture). N2 is now the wild-type strain of reference for all research in *C. elegans* and is still cultured monoxenically on *E. coli* as standard. Thus, laboratory nematodes have been maintained without their native microbiota for some considerable time and almost all work on this organism has been done in this essentially microbiota-free condition.

Surprisingly little is known about the ecology of *C. elegans* in its natural habitat but it certainly differs from laboratory conditions in the range of microorganisms that are encountered. Moreover, when environmentally isolated bacteria are studied in the *C. elegans* laboratory model their effects on the life history of the worm are often profound [2]. It has been increasingly recognized that the worm microbiota is an important consideration in achieving a naturalistic experimental model in which to study, for instance, host–pathogen interactions or worm behavior.

**Fig. 1.**
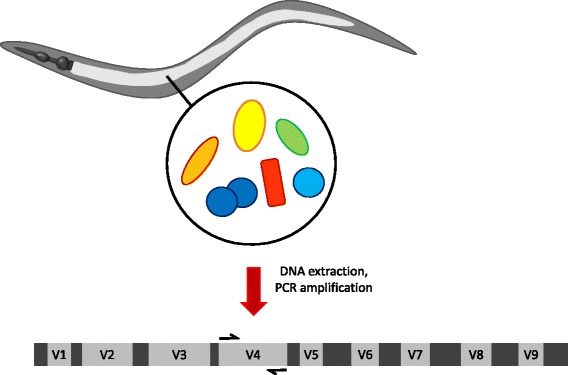
Sequencing bacterial 16S rRNA genes from the worm gut. Adult *C. elegans* showing pharyngeal structures (*dark grey*) and gut (*light grey*) containing live microorganisms. The pharyngeal grinder disrupts *E. coli* cells under most laboratory conditions but in natural environments many microbes survive passage through the pharynx. Total DNA is extracted and the V4 variable region of the 16S ribosomal RNA gene is amplified by PCR prior to sequencing

Dirksen et al [3] present the first step towards understanding understanding the complex interactions of the natural worm microbiota by reporting a 16S rDNA-based “head count” of the bacterial population present in wild nematode isolates (Fig. 1). Interestingly, it appears that nematodes isolated from diverse natural environments—and even those that have been maintained for a short time on *E. coli* following isolation—share a “core” host-defined microbiota. This finding is in agreement with work by Berg et al. [4], who examined the *C. elegans* gut microbiota assembled under laboratory conditions and concluded that host factors play a major role in shaping the bacterial community. Despite differences in how the microbiota under study was established, the “core” *C. elegans* microbiota identified by both groups is similar, suggesting that this core biota can be derived from multiple divergent environments and, importantly for experimentalists, can be established and maintained consistently in the laboratory.

## Genetics

The *C. elegans* genome sequence was completed in 1998—the first multicellular organism for which this was achieved [5]—and has been complemented by previous and subsequent comprehensive genetic studies. The result is a very detailed genetic map in which numerous gene functions have been defined and elucidated [6]. In spite of this, many genes have yet to be assigned a phenotypic function despite attempts to characterize them and this may be due in large part to the monoxenic laboratory conditions under which *C. elegans* is studied. One study which examined the effect of three environmentally isolated bacterial strains on gene expression in *C. elegans* identified 204 unique differentially expressed genes, of which 23 % (the largest fraction) were genes of unknown function [2]. The same study identified possible roles for cuticle synthesis and heat shock protein-encoding genes in the context of host–microbe interactions, suggesting that examining *C. elegans* in different biological contexts may help to elucidate known genetic pathways in the worm.

There is also some intriguing evidence to suggest that not only do genes of unknown function play a role in host–microbe interactions but that some *C. elegans* proteins can be directly activated by bacterial metabolites obtained through commensalism. Gusarov et al. [7] found that soluble guanylyl cyclases in *C. elegans* neurons could be activated by nitric oxide produced by *Bacillus subtilis* in the gut, ultimately leading to extension of worm lifespan (again, interestingly, via heat shock proteins). Such crosstalk between gut biota and worm has interesting implications across a number of life-history traits such as ageing and lifespan. The natural worm microbiota also likely plays a role in pathogen protection, both by directly inhibiting gut colonization by invading pathogens and by regulating innate immune responses in the worm in a manner similar to that described in mammalian models [8].

## Ecology

**Fig. 2.**
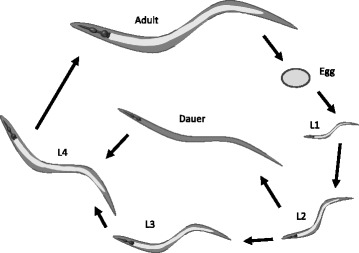
The *C. elegans* lifecycle. Eggs hatch and go through four larval stages (L1–L4) before adulthood. An alternative non-feeding larval stage (*Dauer*) can be initiated at the L2 moult in conditions of high population density, temperature, or starvation and worms can also arrest at the L1 larval stage under starvation conditions. Adapted from [6]

A major natural habitat of *C. elegans* is rotting vegetation and decaying fruit and, like the closely related *Caenorhabditis briggsae* and *Caenorhabditis remanei*, the species has a broad global distribution across temperate and sub-tropical regions. *C. elegans* appears to have a “boom and bust” lifecycle (Fig. 2) and worms in the wild probably spend a large amount of time as dauers, an alternative non-feeding larval stage induced by high population density and starvation in which worms are comparatively resistant to environmental stress and can undergo dispersal to new food sources [9]. The typical microbiota that might be found in either dauers or proliferating populations is not well characterized and might vary in both abundance and composition depending on the resources (for example, seasonal windfalls of fruit) that are available [4]. Examples of soil bacteria previously isolated in association with nematodes include *Micrococcus luteus*, *Bacillus megaterium*, and *Pseudomonas* spp. [2].

Another important aspect of the work by Dirksen et al. [3] is its inclusion of *C. remanei* isolates. *C. remanei* is found in similar habitats and distribution to *C. elegans* but reproduces sexually and, probably as a consequence, has a greater genetic diversity [6, 10]. The existence of a defined “*C. remanei*-type” microbiota is interesting in that it suggests that environment and within-species host diversity are less important than between-species diversity in defining the core microbiota. It is possible that a few, as-yet-undefined host factors are sufficient to define the bacterial community structure of the gut. Like *C. elegans*, *C. remanei* is habitually cultured monoxenically in the lab and this study represents a significant expansion of knowledge regarding its natural microbiota.

## Embracing diversity

From a dearth of information regarding the *Caenorhabditis* microbiome there is now the foundation of an understanding, with two papers characterizing laboratory-established [4] and natural microbiota [3] of *C. elegans* and related taxa. These papers show evidence of the microbiota’s impact on growth, fecundity, and pathogen resistance; this may be complemented in the future by studies on worm behavior.

More work remains to be done to establish the stability of the microbiota over the lifetime of the individual animal, across generations, and in the face of environmental perturbation. Deep sequencing may shed light on gene- and transcript-level diversity of the microbiota and it is interesting to speculate on the host factors and within-community interactions which might drive the transition of bacterial community structure from environment to host.

Although previous studies have attempted to characterize natural interactions of *C. elegans* and bacteria, they are for the most part based on a rather artificial laboratory model, in which worms that are naïve to anything except *E. coli* are suddenly exposed to a huge inoculum of the test organism. Subtle, microbe-specific immune responses and behaviors may be missed amid the noise of “culture shock”. In this respect, a properly characterized microbiota which can be established in the laboratory could be invaluable in separating genuine pathogen response from experimental artefact.

In the *C. elegans* model, researchers have a ready-made environment in which to systematically explore the effect of host factors on the microbiota and vice versa. While it might seem intimidating to introduce such complexity to this heretofore straightforward laboratory model of host–microbe interactions, the time now seems ripe to embrace natural variation. As this study shows, we now have the tools to take on this variation and turn it from a complication into an asset.
